# Classification of occlusal caries severity using spectrophotometric CIELAB measurements and machine learning algorithms

**DOI:** 10.1007/s00784-026-06911-x

**Published:** 2026-05-14

**Authors:** Farah Rashid, James Dudley

**Affiliations:** Adelaide Dental School, Adelaide University, Adelaide, South Australia Australia

**Keywords:** Caries prediction, Occlusal caries, CIELAB, Spectrophotometer, Machine learning, Preventive dentistry

## Abstract

**Objectives:**

This study evaluated whether spectrophotometric CIELAB color measurements can support machine-learning (ML) classification of occlusal caries severity based on ICDAS categories.

**Methods:**

Three hundred extracted human teeth were visually classified using ICDAS (0–4) and served as labels for supervised learning. Five occlusal sites per tooth were measured using a spectrophotometer under standardized conditions. Site-level CIELAB values were transformed into engineered color features and aggregated at the tooth level. Teeth were categorised as sound (ICDAS 0), initial carious lesions (1–2), and moderate carious lesions (3–4). Five ML models: Random Forest (RF), XGBoost, CatBoost, Multilayer Perceptron (MLP), and a Deep Sets architecture were trained. Performance was evaluated using accuracy, balanced accuracy (BA), and macro-F1, with additional binary analyses for early (ICDAS 1–4 vs. 0) and operative lesion detection (ICDAS 3–4 vs. 0–2), based on the included ICDAS range. Learning curve analysis was performed to evaluate the effect of training data size on model performance.

**Results:**

Deep Sets achieved the highest multiclass performance (BA = 0.89) followed by the MLP (BA = 0.72). Tree-based models demonstrated lower performance overall. For early lesion detection, all models showed high sensitivity (SE) but reduced specificity (SP). At the operative level, the MLP achieved 100% SE and moderate SP. Learning-curve analysis showed that neural models benefited most from increased training data, whereas tree-based models showed limited improvement.

**Conclusion:**

Under controlled in-vitro conditions, spectrophotometric CIELAB measurements enabled machine-learning classification of occlusal caries severity. Deep sets achieved the highest overall performance, supporting the potential of color-based approaches as adjunctive tools for caries assessment.

**Clinical relevance:**

Spectrophotometric tooth color measurements may support machine learning classification of occlusal caries severity under controlled conditions, using an image-independent approach. Such color-based methods may complement visual assessment by providing a more standardized and reproducible evaluation of enamel changes following further validation.

**Supplementary Information:**

The online version contains supplementary material available at 10.1007/s00784-026-06911-x.

## Introduction

Dental caries is among the most prevalent chronic conditions, with untreated permanent lesions affecting over 10% of the global population and ranking as a leading cause of oral health burden [1]. Early detection is essential, as initial demineralization can be arrested or reversed through preventive measures before irreversible damage occurs [2]. Visual tactile inspection remains the primary method for occlusal caries assessment and performs reliably for most lesion stages; however, distinguishing initial lesions (ICDAS 1–2) can be challenging due to subtle surface changes and examiner related variability. Radiographs provide additional information but have limited sensitivity for early and non-cavitated lesions along with the risk of radiation exposure [3, 4]. 

Changes in tooth color are among the earliest clinically observable indicators of enamel demineralization. Early lesions may present as white spot opacities or brownish discoloration prior to structural breakdown [5]. Several optical diagnostic devices, including fluorescence-based cameras, near infrared transillumination, optical coherence tomography, and intraoral scanners, have been developed to support clinicians, yet their high cost, technique sensitivity, and limited accessibility restrict routine use in general dental practice [6, 7]. Importantly, many of the devices do not directly quantify tooth color, despite chromatic change being a simple and non-invasive indicator of early enamel alteration [8, 9]. 

Spectrophotometry provides device standardized and reproducible measurement of tooth color in CIELAB color space, allowing sensitive detection of subtle optical changes in enamel [10]. Although widely used for dental shade matching [11], its application for objective assessment of early caries remains unexplored. In contrast, numerous image-based artificial intelligence and machine learning (AIML) studies using intraoral, smartphone or radiographic images have demonstrated promising performance [12–18]. However, their outputs are influenced by lighting, angulation and hardware variability, which may affect reproducibility and limit generalizability in clinical settings [18, 19]. Emerging modalities such as fluorescence and hyperspectral imaging have further advanced AIML research in caries assessment [4, 13–15, 20]. Nevertheless, most existing AIML approaches rely on pixel-based images rather than calibrated numeric color measurements.

Machine learning approaches in dental diagnostics include tree-based models, such as Random Forest (RF), gradient boosting methods (XGBoost and CatBoost), as well as neural networks. These models are well suited to analysing structured numerical data and can identify complex patterns that may not be apparent through conventional analysis [16]. Tree-based models are generally effective for tabular data and can capture non-linear relationships, whereas neural networks are capable of modelling more complex feature interactions and may better capture subtle patterns in high dimensional data. However, most approaches treat each tooth as a single set of measurements and may not fully capture variation across multiple sites within a tooth. More recent modelling approaches are designed to better represent such multi-site information within a single sample, including architectures such as Deep Sets [21]. While spectrophotometry enables objective quantification of tooth color, its relationship with caries severity remains underexplored. Accordingly, rather than positioning color measurement as a standalone clinical tool, this present study adopts an exploratory approach to evaluate whether CIELAB parameters contain sufficient discriminatory information to support machine-learning classification under controlled conditions.

To date, no study has evaluated whether spectrophotometric CIELAB color parameters alone, independent of photographic or radiographic inputs, can support machine learning (ML) classification of caries severity. To address this research gap, the present study developed and compared multiple AIML models trained exclusively on spectrophotometric CIELAB measurements obtained from five occlusal sites per tooth. Teeth were classified as sound (ICDAS 0), initial carious lesions (ICDAS 1–2) and moderate carious lesions (ICDAS 3–4). By analysing objective color parameters collected under controlled in-vitro conditions, this study aimed to determine whether numeric CIELAB features provide sufficient information for automated classification of occlusal caries severity. It was hypothesized that spectrophotometric CIELAB color parameters alone would enable machine-learning classification of occlusal caries severity across ICDAS categories.

## Methods

### Study design

**Fig. 1:**
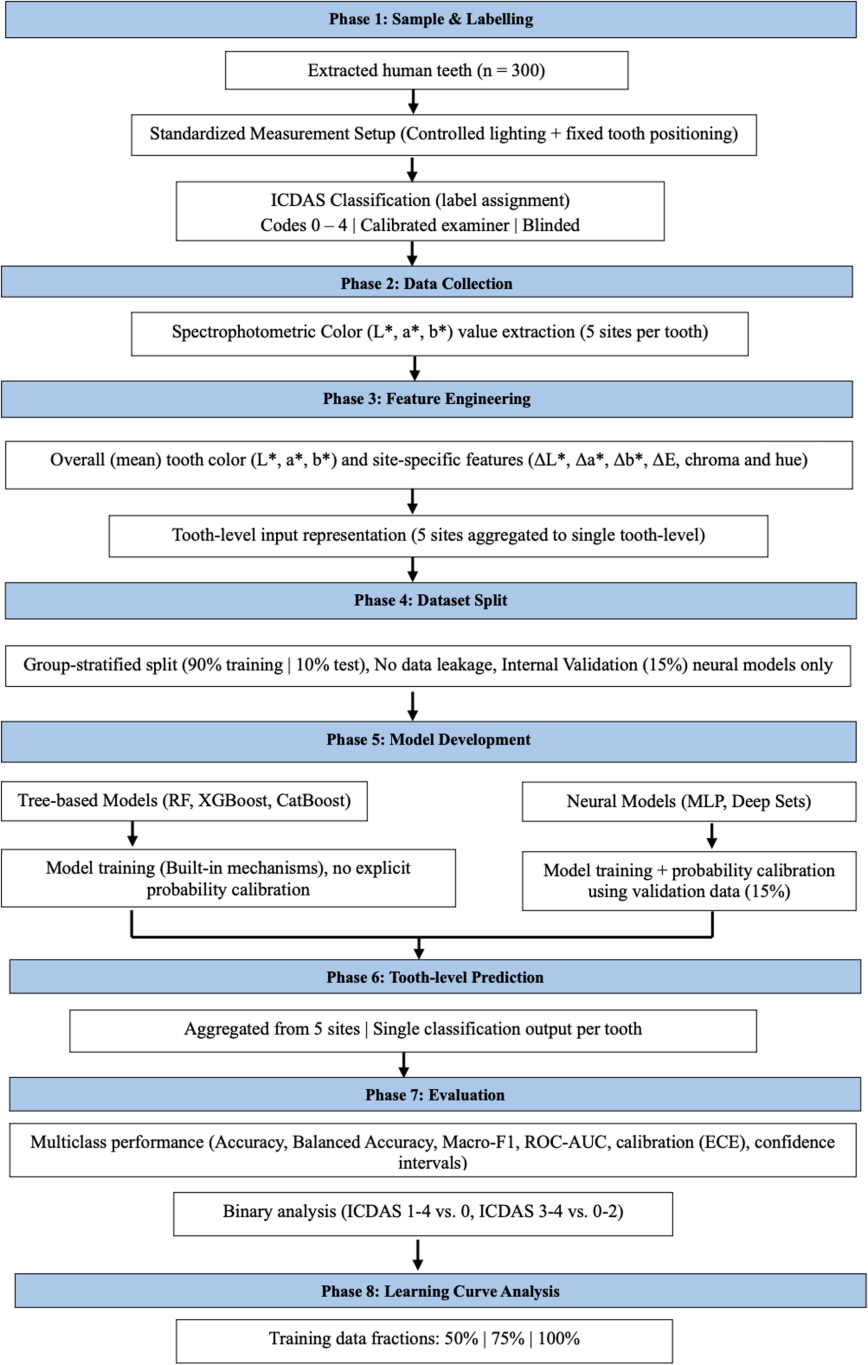
Study workflow

This cross sectional in-vitro study was conducted using de-identified extracted human teeth (*n* = 300) collected from the Adelaide University Dental Simulation Clinic. The reporting of this study was guided by Transparent Reporting of a Multivariable Prediction Model for Individual Prognosis or Diagnosis (TRIPOD and TRIPOD-AI) principles [22], with consideration of relevant Standards for Reporting of Diagnostic Accuracy Studies (STARD 2015) elements [23]. Formal ethics approval was not required under institutional guidelines for anonymized human dental tissues. The in-vitro approach enabled precise control of environmental lighting, geometry, and surface moisture, which are known to influence optical color readings. The study workflow is presented in Fig. 1.

All analyses were conducted in Python programming language (Version 3.10) using scikit learn (for classical models), XGBoost and CatBoost (for gradient-boosting ensembles), and TensorFlow Keras (for deep learning models). All experiments were executed in Google Colab (Google LLC, Mountain View, CA, USA) on an NVIDIA Tesla T4 GPU (NVIDIA Corporation, Santa Clara, CA, USA). A fixed random seed (42) was applied across NumPy, scikit-learn, and TensorFlow to ensure reproducibility by maintaining consistent data splitting and model initialization across runs.

### Standardization of lighting and specimen positioning

ICDAS scoring and spectrophotometric color measurements were performed within a standardized PhotoBox with neutral-grey background and controlled illumination (1000–1500 lx at 5100–5500 K) to simulate daylight conditions [24]. Each tooth was mounted with the occlusal surface facing the examiner and oriented perpendicular to the measurement axis and stabilized using non-reflective soft clay. Distances were maintained at 150 mm from the platform, 200 mm from the light source and 500 mm from the examiner. The experimental setup was fixed throughout the data collection process to ensure that these distances remained constant across all specimens. These parameters were determined through pilot testing to minimize shadow formation, specular reflection and angular illumination artifacts while maintaining consistent probe alignment and visual assessment conditions across all specimens. A visual representation of this experimental setup that used for ICDAS label assignment and spectrophotometric data collection is provided in Supplementary Fig. 1.

### Sample characteristics and ICDAS label assignment

Extracted permanent molars and premolars with ICDAS scores ranging from 0 to 4 were included, representing sound teeth (ICDAS 0), initial carious (ICDAS 1–2) and moderate carious (ICDAS 3–4) occlusal condition. A representative image of the samples that used for this analysis is presented in Supplementary Fig. 2. The study included both molars and premolars to represent posterior occlusal surfaces. Stratification by tooth type was not performed, as the study aimed to evaluate posterior teeth collectively rather than compare molars and premolars as separate group. Each tooth received a single ICDAS score based on visual examination of the entire occlusal surface and only teeth with a uniform ICDAS category were included to ensure that all sampled sites reflected the same severity level. Teeth with extensive cavitation (ICDAS 5–6), proximal caries, or non-carious surface loss (abrasion, erosion, attrition) were excluded to minimise confounding optical changes unrelated to demineralization.

ICDAS scoring was performed by a registered dentist using the ICDAS decision tree [25] and the examiner was blinded to both spectrophotometric color data and machine learning model outputs. Examiner calibration was achieved through completion of the ICCMS™ e-learning module, followed by two independent scoring sessions of 40 randomly selected teeth. Intra-examiner agreement was excellent (Cohen’s K = 0.90 unweighted; 0.96 weighted). ICDAS scores were used solely as categorical labels for supervised machine learning training and were not treated as a diagnostic standard.

A formal statistical power analysis was not performed, as the study focused on supervised machine-learning model development and predictive performance evaluation rather than hypothesis testing within a traditional inferential statistical framework. The sample size (*n* = 300) was determined pragmatically based on the availability of extracted teeth and is consistent with or larger than datasets used in comparable in-vitro AI studies. All teeth were disinfected using 10% neutral buffered formalin solution (Thermo Fisher Scientific’s, Waltham, MA, USA) for 10 days, thoroughly rinsed and stored in distilled water at room temperature to preserve enamel color and microhardness [26]. 

### Spectrophotometric color data collection

Spectrophotometric CIELAB measurements were obtained using a VITA Easyshade V spectrophotometer (VITA Zahnfabrik GmbH, Bad Sackingen, Germany), operated by a single trained examiner with over four years of experience using the device for research purposes. Prior to data collection, the examiner was familiarized with the measurement protocol through repeated practice measurements to ensure consistency in probe positioning and data acquisition. All measurements were performed under standardized conditions to minimize operator related variability. The spectrophotometer was recalibrated before each measurement according to the manufacturer’s instructions. Measurement reliability was supported by strict standardization of the color acquisition protocol, including controlled lighting, fixed geometry and consistent probe positioning across all specimens. The probe tip was positioned perpendicular to the enamel surface and placed in light contact with the tooth surface without applying additional pressure, thereby maintaining a constant working distance and ensuring consistent measurement geometry across specimens. These measures are consistent with established protocols, as the intra-device repeatability and reliability of dental spectrophotometers for CIELAB color measurement have been well documented in previous studies [11, 27, 28]. 

Five anatomically standardized occlusal sites were measured for each tooth, including two buccal cusp slopes, two mesial cusp slopes and the central pit, selected based on consistent anatomical landmarks to capture intra-tooth color variation while ensuring reproducible sampling across the specimens. In total, 1500 spectrophotometric measurements (5 sites × 300 teeth) were collected under controlled conditions. Although multiple sites were measured per tooth, these measurements were not treated as independent samples. Instead, they were used collectively as inputs during model training to capture intra-tooth variability, with predictions subsequently aggregated to generate a single tooth-level classification outcome.

### Machine-learning models

Five supervised learning models were developed to classify caries severity based on numeric color features: three tree-based ensembles (Random Forest, XGBoost and CatBoost) and two neural network architectures (Multilayer Perceptron, and a Deep Sets model). These models were selected because they are well suited for structural tabular data and can capture nonlinear relationships in color patterns associated with demineralization [4, 17]. Tree based models were selected for their strong performance on biomedical datasets, and relative interpretability [13], while Neural models were selected to capture higher-order feature interactions and to accommodate multisite structure of the spectrophotometric measurements [29]. Full architectural specifications, training procedures, and hyperparameter settings are provided in Supplementary Table 1.

### Feature engineering and data pre-processing

Each tooth was treated as a single analytical unit comprising five spectrophotometric measurement sites. The overall tooth color was first defined using the mean L* (lightness), a* (green – red) and b* (blue - yellow) values. To better reflect the clinical presentation of early caries, additional features were derived to capture how each site differed from the overall tooth color. This approach emphasizes localized color variation, as early enamel demineralization typically presents as subtle site-specific changes rather than uniform alterations across the tooth surface.

Additional color descriptors, including chroma (color intensity), hue (color shade), and overall color difference (ΔE), were calculated to further characterize these variations. Site-specific differences from the overall tooth color were also quantified using ΔL* (change in lightness), Δa* (change along with the green-red axis), Δb* (change the blue-yellow axis). Color differences (ΔE) were evaluated relative to both the individual tooth baseline and the average color of sound teeth (ICDAS 0), providing a clinically relevant reference for comparison.

The five site-level measurements were combined by deriving both overall tooth color (mean L*, a* and b*) and site-specific deviations (ΔL*, Δa*, Δb*, ΔE, chroma and hue), allowing both global and local color characteristics to be represented. These features-engineered variables, derived from the site-level spectrophotometric measurements (1500 observations), were used as inputs to the machine-learning models, enabling incorporation of information from all five sites while producing single classification outcome for each tooth.

All feature calculations (mean L*, a*, b*, ΔL*, Δa*, Δb*, chroma, hue, and ΔE) were derived using established colorimetric formulas (Supplementary Table 2) commonly applied in dental color science, alongside study-specific transformations designed to capture intra-tooth variation. Detailed descriptions of feature calculations and model specific preprocessing steps are provided in the Supplementary Table 2.

### Dataset split

To prevent information leakage and ensure unbiased model evaluation, all data splitting was performed at the tooth level, such that all five spectrophotometric measurement sites from the same tooth were retained within the same dataset partition. A group stratified splitting approach (group = tooth ID) was used to preserve the proportional distribution of the three diagnostic categories (sound, initial, moderate) across the training, validation and test sets, while ensuring that measurements from the same tooth were not split across different subsets.

The dataset comprised 300 teeth, including 40 sound, 181 initial and 79 moderate carious lesions. A 90% −10% split was applied to maximise the amount of data available for model training while retaining an independent test set for unbiased evaluation, resulting in a test set of approximately 30 teeth (10% of the dataset) with class proportions maintained through stratification. Based on the stratified split, the test set contained approximately 4 sound, 18 initial and 8 moderate teeth, reflecting the overall dataset distribution.

For the classical tree-based models (RF, XGBoost, CatBoost), the dataset was split into 90% training and 10% testing. For the MLP and Deep Sets architectures, the same split was applied, with 15% of the training portion further allocated as an internal validation set to support model selection, hyper-parameter optimization and probability calibration.

All preprocessing steps, including feature engineering, normalization and calibration, were conducted exclusively on the training data, and the test set was used only once for the final performance evaluation to ensure an unbiased and independent assessment. A hold-out test approach was selected to ensure a strictly independent evaluation at the tooth level and to minimize the risk of information leakage, particularly given that multiple measurements were obtained from each tooth.

To mitigate the impact of class imbalance, stratified sampling was combined with model-specific strategies, including approaches that increased the contribution of underrepresented classes during training (e.g., class weighting for tree-based models and focal loss for neural network models). By design, this splitting approach ensured that no tooth appeared in more than one subset, thereby maintaining strict independence between training, validation and test partitions.

### Model training and prediction generation

All models were trained on the same dataset using strategies designed to address class imbalance and to minimize overfitting, thereby improving the ability of the models to accurately classify caries severity in independent test samples not used during model training. The test set was reserved exclusively for final evaluation.

For neural network models, a validation subset was derived from the training data to support model tuning, early stopping and probability calibration. Regularization techniques such as dropout, Gaussian noise injection, and adaptive learning-rate adjustment, were applied to improve model stability and reduce sensitivity to noise. For tree-based models, training relied on inherent model mechanisms, including learning-rate control, L2 regularization, subsampling, and ensemble-based approaches. Internal validation procedures were used during training, but no separate validation subset was used for probability calibration. Probability calibration was performed for neural network models using the validation data to improve the reliability of predicted probabilities. In contrast, tree-based model outputs were not explicitly calibrated; instead, their predictions were refined using model-specific training strategies and post-processing approaches. For all models, predictions were generated at the tooth level by integrating information from the five measurement sites associated with each tooth, ensuring that classification reflected overall lesion characteristics rather than individual site measurements.

### Evaluation protocol

Model performance was assessed exclusively on the independent test set, which remained separate from all training, hyperparameter tuning, and calibration procedures. The primary evaluation examined multiclass classification performance across predefined ICDAS categories (sound, initial, moderate), summarized using accuracy, balanced accuracy (BA) and macro-F1.

To reflect clinically relevant decision-making scenarios, two additional label-based classification contrasts were evaluated to examine model behaviour: early lesion detection (ICDAS 1–4 vs. 0) and operative level detection (ICDAS 3–4 vs. 0–2). These contrasts were used to assess model performance at clinically meaningful thresholds and were not intended to redefine diagnostic criteria. For each contrast, receiver operating characteristic (ROC) curves and the corresponding area under the curve (AUC) were computed, along with ΔAUC values relative to the best-performing model. Multiclass AUC was calculated using one-vs-rest (OVR) approach.

Calibration performance was assessed using Expected Calibration Error (ECE) and calibration curves before and after scaling. Confidence intervals for all performance metrics (accuracy, SE, SP and AUC) were estimated using clustered bootstrapping with 1,000 iterations, accounting for repeated site-level measurements within each tooth. Pairwise comparisons were performed using McNemar’s test for accuracy, DeLong’s test with Holm correction for AUC differences, and Wilcoxon signed-rank tests for paired metric comparisons where appropriate. All statistical tests were two-sided, and a significance level of *p* < 0.05 was applied. As this study focused on model discrimination under controlled in vitro conditions rather than clinical diagnostic performance, prevalence-dependent measures such as predictive values were not included, as these measures are highly dependent on disease prevalence and may not generalize beyond the experimental dataset.

### Learning curve analyses

Each model was trained using 50%, 75%, and 100% of the available training set while maintaining identical preprocessing steps, class weighting, hyperparameters and evaluation procedures. Tooth-level accuracy, BA, macro-F1, and Macro-AUC and model performance improvement from 100% (Δ) were recorded for each subset to characterise how effectively each model learned from smaller vs. larger datasets. All procedures used for generating tooth level predictions were applied consistently across all dataset sizes, as described in the primary analysis.

## Results

A total of 1500 spectrophotometric site level color measurements were collected from 300 extracted teeth. These measurements were grouped into three ICDAS categories assigned at the tooth level (sound, initial, and moderate). The distribution of teeth and measurement points across categories is summarized in Table 1.

**Table 1:** Dataset characteristics (tooth and measurement-level summary)

ICDAS labels	Teeth (*n*)	% of total teeth	Color Measurement sites (*n* = teeth × 5)	% of total measurement points
Sound (0)	40	13.3%	200	13.3%
Initial (ICDAS 1 to 2)	181	60.3%	905	60.3%
Moderate (3 to 4)	79	26.3%	395	26.3%
Total	300	100%	1500	100%

ICDAS categories were assigned at the tooth level, and each tooth contributed 5 spectrophotometric measurement sites. ICDAS labels were used solely as classification targets for the machine learning models

### Performance of the machine learning models for three-class caries classification

Model performance was evaluated using accuracy, macro-F1 score and balanced accuracy (BA), which was reported as a primary metric due to the class imbalance in the dataset (Table 1). Performance varied substantially across the models depending on the underlying architecture. The Deep Sets model achieved the strongest overall classification performance across ICDAS categories, showing the highest balanced accuracy [BA = 0.890 (95% CI = 0.750–0.900)] and macro-F1 score [0.730 (95% CI = 0.442–0.900)], indicating superior ability to correctly assign teeth to sound, initial, and moderate ICDAS categories. The MLP also demonstrated comparatively strong classification performance [BA = 0.720 (95% CI = 0.600–0.867)], although its class-wise performance was less consistent across categories compared with the Deep Sets model.

**Fig. 2:**
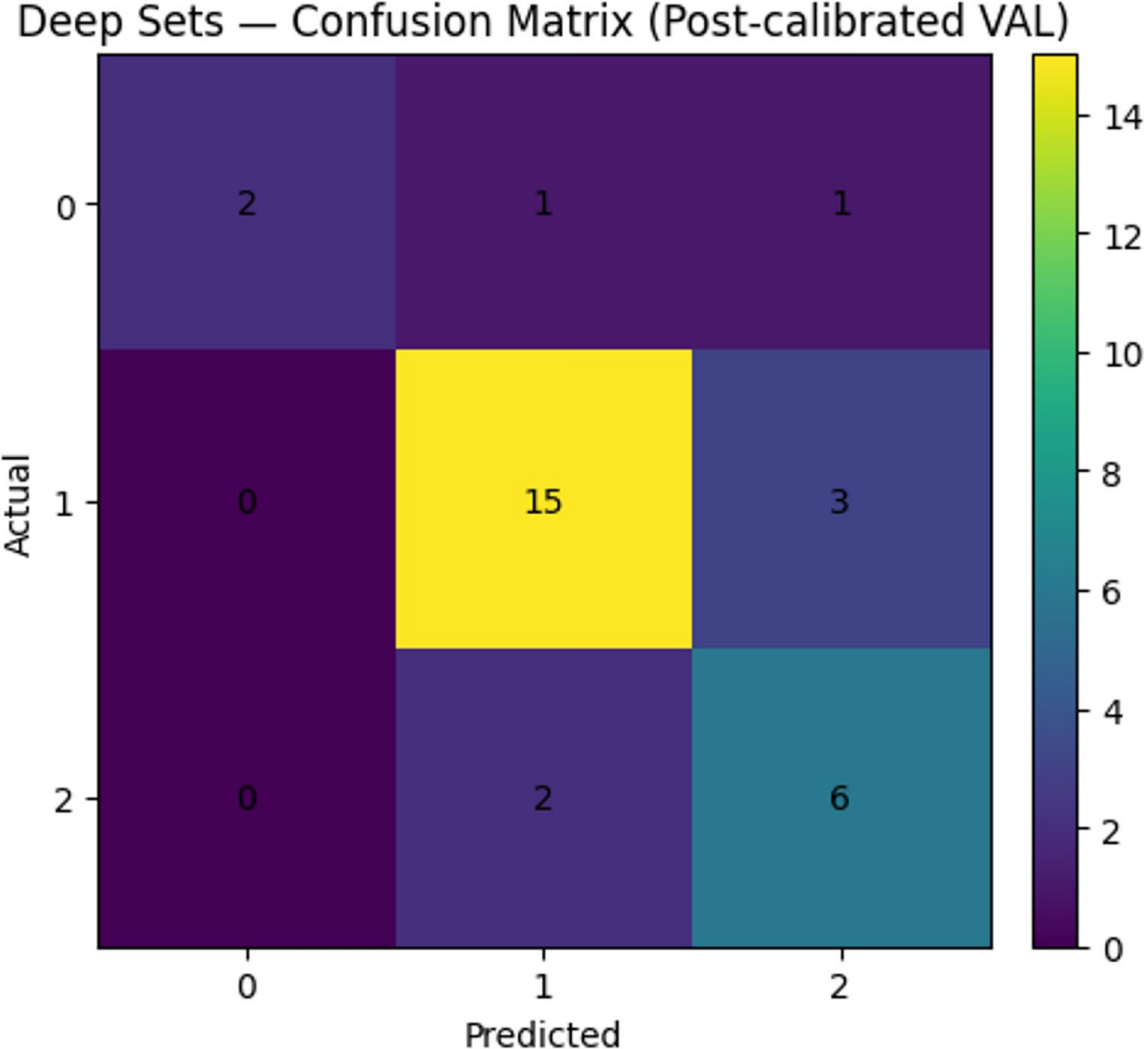
Confusion matrix for the Deep Sets model on the independent test set, showing tooth level classification performance across the three ICDAS categories (0 = sound, 1 = initial, 2 = moderate)

Among the tree-based methods, CatBoost consistently outperformed both XGBoost and Random Forest, while RF showed the lowest overall performance [BA = 0.383 (95% CI = 0.029–0.394) and macro-F1 = 0.341 (95% CI = 0.288–0.397)]. These findings indicate that while several models were able to learn meaningful patterns from the spectrophotometric data, the Deep Sets architecture was most effective for this three-class classification task. Complete multiclass performance metrics for all models are summarised in Table 2. Class wise prediction patterns for the best performing model are visualized in the confusion matrix in Fig. 2, highlighting typical misclassifications among sound, initial and moderate categories.

**Table 2:** Tooth-level classification performance of all machine learning models

Model	BA (95% CI)	Macro-F1 (95% CI)	SE (Per class Recall)	SP (True negative rate)	AUC (95% CI)
Random Forest	0.383 (0.029–0.394)	0.341 (0.288–0.397)	33.8%	83.6%	0.690 (0.644–0.720)
XGBoost	0.487 (0.367–0.678)	0.497 (0.467–0.589)	58.6%	75.2%	0.705 (0.634–0.756)
CatBoost	0.532 (0.466–0.600)	0.515 (0.455–0.573)	53.2%	74.3%	0.765 (0.691–0.885)
MLP	0.720 (0.600–0.867)	0.690 (0.472–0.867)	72.0%	86.0%	0.800 (0.685–0.987)
Deep Sets	0.890 (0.750–0.900)	0.730 (0.442–0.900)	70.0%	81.8%	0.782 (0.643–0.921)

*BA* = Balanced Accuracy, *AUC = *Area Under theCurve, *SE* = Sensitivity, *SP =* Specificity. SE and SP are reported as classification metrics and reflect agreement with ICDAS categories rather than diagnostic accuracy against a reference standard

### Model performance across ICDAS based binary classification contrasts

For early classification (ICDAS 1–4 vs. 0), all models showed high SE [89.2% (95% CI = 0.854–0.927) to 100% (95% CI = 1.000–1.000)] but comparatively lower SP [12.5% (95% CI = 0.027–0.233) to 50% (95% CI = 0.350–0.650)], reflecting a tendency to assign a greater portion of teeth to the ICDAS 1–4 category. Deep Sets achieved the most balanced performance [BA = 0.750 (95% CI = 0.500–1.000)], while the MLP and RF maintained high SE [96.2% (95% CI = 0.885–1.000) to 96.9% (95% CI = 0.946–0.988)] but substantially lower SP [25% (95% CI = 0.000–0.750) and 12.5% (95% CI = 0.027–0.233), respectively]. The tree-based models (XGBoost and CatBoost) exhibited a more conservative classification pattern, producing fewer assignments of sound teeth to the ICDAS 1–4 category, but misclassifying many teeth labelled ICDAS 1–4.

For the operative level classification (ICDAS 3–4 vs. 0–2), the MLP model demonstrated the strongest balance between SE [100% (95% CI = 1.000–1.000)] and SP [72.7%, 95% CI = 0.545–0.909) within the study dataset, followed by Deep Sets SE [75% (95% CI = 0.400–1.000) and SP [81.8% (95% CI = 0.636–0.958)]. The tree-based models tended to prioritise SP [84.2% (95% CI = 0.792–0.887) to 87.8% (95% CI = 0.833–0.918)] over SE [38% (95% CI = 0.275–0.486) to 54.4% (95% CI = 0.430–0.658)], resulting in more frequent misclassification of teeth within the ICDAS 3–4 category. Full performance metrics for both contrasts are summarised in Table 3.

**Table 3:** Performance of machine learning models for two ICDAS based binary classification contrasts (ICDAS 1–4 vs. 0 and ICDAS 3–4 vs. 0–2)

Model Name	SE (Per class Recall) (95% CI)	SP (True negative rate) (95% CI)	BA (95% CI)	AUC (95% CI)	ΔAUC vs. best	SE (Per class Recall) (95% CI)	SP (True negative rate) (95% CI)	BA (95% CI)	AUC (95% CI)	ΔAUC vs. best
		Contrast 1: ICDAS 1–4 vs. 0 (Early lesions detection)						Contrast 2: ICDAS 3–4 vs. 0–2 (Operative level detection)		
Random Forest	96.9% (0.946–0.988)	12.5% (0.027–0.233)	0.547 (0.498–0.604)	0.758 (0.688–0.825)	− 0.017	38% (0.275–0.486)	87.8% (0.833–0.918)	0.629 (0.573–0.686)	0.722 (0.657–0.781)	−0.019
XGBoost	92.3% (0.880–0.954)	40% (0.250–0.550)	0.662 (0.584–0.738)	0.825 (0.761–0.883)	− 0.038	54.4% (0.430–0.658)	86.4% (0.814–0.905)	0.704 (0.644–0.764)	0.797 (0.740–0.849)	−0.073
CatBoost	89.2% (0.854–0.927)	50% (0.350–0.650)	0.696 (0.615–0.775)	0.808 (0.741–0.868)	− 0.009	54.4% (0.430–0.658)	84.2% (0.792–0.887)	0.693 (0.632–0.753)	0.797 (0.739–0.849)	−0.045
MLP	96.2% (0.885–1.000)	25% (0.000–0.750)	0.606 (0.462–0.856)	0.808 (0.538–1.000)	0.000	100% (1.000–1.000)	72.7% (0.545–0.909)	0.864 (0.773–0.955)	0.841 (0.688–0.960)	−0.074
Deep Sets	100% (1.000–1.000)	50% (0.000–1.000)	0.750 (0.500–1.000)	0.798 (0.482–1.000)	− 0.019	75% (0.400–1.000)	81.8% (0.636–0.958)	0.784 (0.586–0.940)	0.824 (0.645–0.963)	0.000

All metrics were computed at the tooth level after aggregating predictions from 5 spectrophotometric measurement points per tooth. Confidence intervals (CI) were obtained by clustered bootstrapping. *SE* = Sensitivity, *SP* = Specificity, *BA* = Balanced Accuracy, *AUC* = Area Under Curve and ΔAUC values represent the difference between each model’s AUC and the highest AUC at the corresponding label. SE and SP are reported as classification metrics reflecting agreement with ICDAS labels and should not be interpreted as diagnostic accuracy against a reference standard

**Fig. 3:**
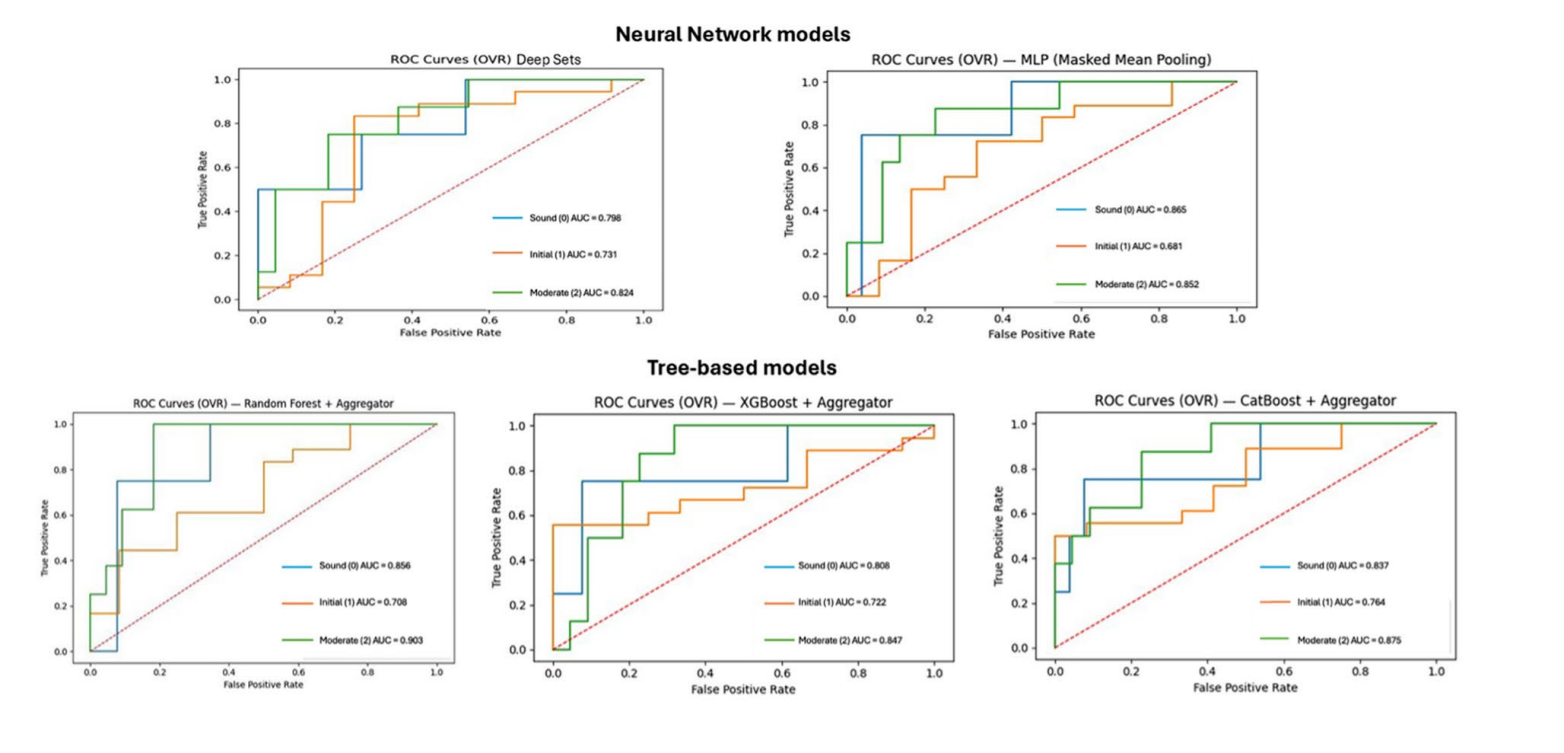
Multiclass One-vs-Rest ROC curves for five machine learning models on the independent test set. Separate curves are shown for sound (ICDAS 0), initial (ICDAS 1 - 2) and moderate (ICDAS 3 - 4) categories. These curves illustrate threshold independent classification performance and the relative separability of each ICDAS class across models

Figure 3 presents the multiclass one-vs-rest ROC curves, illustrating separability across ICDAS 0, ICDAS 1–2 and ICDAS 3–4 categories. Neural models (Deep Sets and MLP) showed smoother, more distinct ROC trajectories, while the initial category remained the most challenging across architecture. These analyses are presented to characterise model behaviour under different classification scenarios and should be interpreted in the context of the study design.

### Effect of training data fractions on model performance

Across all models, classification performance generally improved as larger fractions of the training data were used, although the magnitude and consistency of these changes varied between models architectures. Deep Sets showed the most pronounced learning pattern, with BA increased from 0.563 (95% CI = 0.439–0.672) at 50% training data to 0.775 (95% CI = 0.684–0.856) at 75% reaching 0.890 (95% CI = 0.750–0.900) with the full dataset. Macro-F1 and Macro-AUC followed similar trends, and the Δ metric indicated the largest relative performance gain at reduced data fractions (+ 0.180 at 50%). Similarly, the MLP demonstrated clear benefit from additional data, with BA increasing from 0.531 (95% CI = 0.455–0.678) at 50% training data to 0.640 (95% CI = 0.574–0.721) at 75% and reaching 0.720 (95% CI = 0.600–0.867) at 100% training data.

In contrast, CatBoost and XGBoost exhibited more modest and less consistent patterns. CatBoost achieved its highest BA at 75% [0.660(95% CI = 0.542–0.828)], while XGBoost showed relatively small variation across all training fractions. RF similarly demonstrated limited improvement and the lowest BA at 100% data [0.383, 95% CI = 0.029–0.394)]. Full results for all models and training fractions are provided in Table 4.

**Table 4:** Effect of training data fraction (50%, 70% and 100%) on tooth level classification performance for the five machine learning models. All metrics computed after aggregating 5 site level measurements per tooth. Confidence intervals clustered by tooth ID

Model	Data Fraction	Accuracy (95% CI)	Balanced Accuracy (95% CI)	Macro-F1 (95% CI)	Macro-AUC (95% CI)	Δ (Model performance improvement from 100%)
Random Forest	50%	0.657 (0.578–0.722)	0.490 (0.426–0.582)	0.473 (0.400–0.614)	0.779 (0.694–0.846)	− 0.090
	75%	0.655 (0.578–0.722)	0.484 (0.412–0.585)	0.462 (0.393–0.580)	0.798 (0.764–0.830)	− 0.088
	100%	0.567 (0.433–0.689)	0.383 (0.029–0.394)	0.341 (0.288–0.397)	0.796 (0.765–0.832)	0.000
XGBoost	50%	0.650 (0.567–0.733)	0.605 (0.452–0.709)	0.588 (0.437–0.687)	0.781 (0.696–0.842)	0.000
	75%	0.610 (0.444–0.700)	0.574 (0.374–0.767)	0.545 (0.376–0.693)	0.798 (0.729–0.838)	+ 0.040
	100%	0.650 (0.555–0.722)	0.487 (0.367–0.678)	0.497 (0.467–0.589)	0.798 (0.729–0.838)	0.000
CatBoost	50%	0.488 (0.311–0.712)	0.623 (0.511–0.759)	0.485 (0.315–0.697)	0.814 (0.771–0.871)	+ 0.129
	75%	0.564 (0.311–0.745)	0.660 (0.542–0.828)	0.549 (0.308–0.743)	0.819 (0.771–0.847)	+ 0.053
	100%	0.617 (0.333–0.700)	0.532 (0.466–0.600)	0.515 (0.455–0.573)	0.823 (0.788–0.841)	0.000
MLP	50%	0.657 (0.578–0.722)	0.531 (0.455–0.678)	0.518 (0.420–0.676)	0.778 (0.729–0.812)	+ 0.119
	75%	0.671 (0.633–0.700)	0.640 (0.574–0.721)	0.526 (0.443–0.659)	0.772 (0.747–0.797)	+ 0.105
	100%	0.776 (0.655–0.895)	0.720 (0.600–0.867)	0.690 (0.472–0.867)	0.764 (0.715–0.808)	0.000
Deep Sets (Attention)	50%	0.650 (0.600–0.700)	0.563 (0.439–0.672)	0.550 (0.412–0.626)	0.766 (0.704–0.811)	+ 0.180
	75%	0.678 (0.567–0.765)	0.775 (0.684–0.856)	0.590 (0.374–0.614)	0.761 (0.687–0.819)	+ 0.115
	100%	0.925 (0.714–0.945)	0.890 (0.750–0.900)	0.730 (0.442–0.900)	0.789 (0.442–0.900)	0.000

All metrics were computed at the tooth level after aggregating predictions from 5 spectrophotometric color measurements per tooth. Confidence intervals were obtained using clustered bootstrapping. *SE = *Sensitivity, *SP* = Specificity, *BA* = Balanced Accuracy, *AUC* = Area Under the Curve, Δ values represent the change in performance relative to the full-data (100%) model for each architecture

**Fig. 4:**
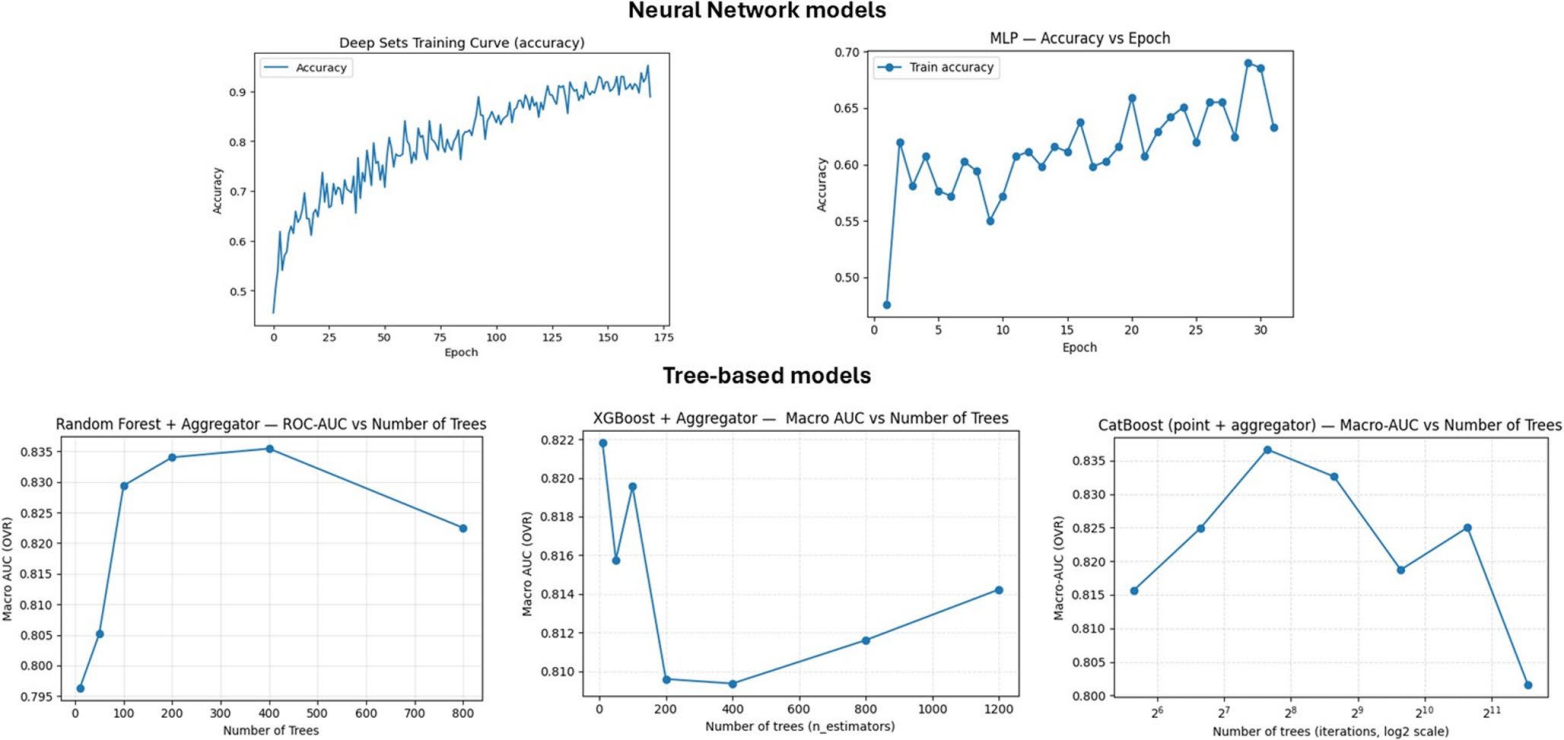
Learning curves for all models as the amount of training data increased. Neural-network (Deep sets, MLP) are shown across training epochs, whereas tree-based models (Random forest, XGBoost, CatBoost) are shown across their number of trees. Balanced accuracy is plotted at the tooth level comparability

These trends are visualised in Fig. 4. Deep Sets exhibited the steepest and most sustained improvement with increasing data, whereas the MLP showed a slower but consistent upward trend. In contrast, the three tree-based models demonstrated early performance saturation, indicating minimal additional benefit from increased data volume relative to the neural architectures.

## Discussion

This study examined whether standardized spectrophotometric CIELAB measurements from multiple occlusal sites can serve as effective inputs for machine learning classification of ICDAS categories. Across the five evaluated algorithms, the color features allowed differentiation between sound, initial and moderate ICDAS classes. Neural network models, particularly Deep Sets, demonstrated the highest overall performance within this dataset, followed by multilayer perception (MLP), whereas tree-based models exhibited comparatively lower balanced accuracy (BA). By relying solely on calibrated color measurements rather than photographic and radiographic inputs, these findings suggest that spectrophotometric data may represent a potential alternative input modality for caries related classification tasks.

The Deep Sets model showed the highest multiclass balanced accuracy within this dataset and performed consistently across both binary classification contrasts, while the MLP showed a slight advantage at the operative-level threshold (ICDAS 3–4 vs. 0–2). This behaviour may reflect the suitability of the CIELAB color space, a device standardised [11, 30] and perceptually uniform system, for capturing subtle optical variations in L*, a*, and b* values associated with enamel demineralization. As the five occlusal measurement sites are unordered, the Deep Sets architecture, which is designed to analyse unordered multisite inputs, is likely well suited to this type of data. By processing each site independently and aggregating them through attention pooling, the model may better capture informative color features and their relative contributions, which may facilitate modelling of intra-tooth heterogeneity. This may be particularly relevant for early-stage lesions, where color changes are often spatially variable [29]. Consistent with this interpretation, Deep Sets also showed the most pronounced improvement with increasing training data **(**Table 4**)**, suggesting that it may benefit from additional spectrophotometric color information.

Previous studies have explored the use of color information for caries detection, primarily through RGB image-based approaches. Studies by Adnan et al. [18], Asci et al. [16], and Navarro et al. [12] have shown that color cues extracted from radiographs, smartphone images or DSLR photographs can support caries detection. However, RGB values are not perceptually uniform and are strongly influenced by imaging conditions such as camera hardware, exposure, settings and in-camera processing [19, 31, 32], and may not directly represent enamel optical properties. In contrast, the CIELAB color system used in this study is device-calibrated and enables computation of clinically interpretable color differences (ΔL*, Δa*, Δb*, and ΔE), which may more closely reflect underlying demineralization dynamics. This difference may have contributed to the improved performance observed with spectrophotometry-based inputs, particularly in models such as Deep Sets, which may be better able to capture subtle and spatially variable color differences.

Comparable trends have been reported in prior deep learning studies using image-based inputs acquired under controlled conditions. Kühnisch et al. [33] reported relatively high AUC values using MobileNetV2 on standardized intraoral photographs, while Yang and Chen et al., [34] achieved multistage ICDAS classification using YOLO-based models following extensive preprocessing. Similarly, Pornprasertsuk-Damrongsri et al. [35] reported accurate tissue-level differentiation using Attention U-Net on radiographs. Although the present study used numerical CIELAB color coordinates rather than image data, it was based on similar principles, including standardized data acquisition, consistent ICDAS labelling, and the use of architectures capable of capturing subtle classification-relevant features across multiple input sites.

Direct comparison with image-based approaches should be made cautiously, as data modalities and acquisition conditions differ substantially. Convolutional neural networks (CNN) operate on high-dimensional image data that can be affected by lighting variability, image artifacts, and soft-tissue interference, often requiring extensive preprocessing and augmentation [33–35]. In contrast, the present study used low-dimensional, device standardized color measurements collected under controlled optical conditions, which may provide a more stable representation of enamel color variation. Rather than analysing pixels-level features, the models learned from multisite color profiles aggregated at the tooth level, suggesting that meaningful patterns may be extracted from compact, quantitatively measured color inputs.

A complementary pattern was observed for MLP. While it demonstrated strong overall classification performance and slightly higher sensitivity and specificity at certain operating points, including achieving 100% sensitivity at the ICDAS 3–4 threshold within the dataset, it exhibited lower balance accuracy compared with Deep sets. This may reflect the impact of the class imbalance in the dataset **(**Table 1**)**, where models with high sensitivity for early lesions may not effectively distinguish between all ICDAS categories [36]. Consequently, balanced accuracy provided a more reliable summary of model behaviour and suggested that the MLP may have greater difficulty in separating intermediate lesion states. This difference in performance reflects both dataset characteristics and architectural design.

The MLP aggregates all five site-level CIELAB measurements into a single representation, which may enhance discrimination when color differences are more pronounced, but may limit its ability to capture stable and spatially variable patterns typical of transitional ICDAS stages [37]. In contrast, the Deep Sets model processes each site independently before aggregation, which may allow more effective modelling of gradual and uneven demineralization across the occlusal surface. Although the MLP achieved slightly higher ROC-AUC values, this did not translate into improved balanced accuracy, as classification errors were more frequent within the intermediate ICDAS categories. Similar observations have been reported in neural network studies applied to CBCT derived ICDAS staging [38]. The relatively wide confidence intervals observed in some comparisons further suggest the need for validation in larger dataset.

Binary classification analyses suggested complementary strengths between the two neural models. Deep Sets showed relatively strong performance for early lesion classification (ICDAS 1–4 vs. 0), maintaining higher specificity than the MLP while preserving high sensitivity, which may indicate improved discrimination of subtle color variations. In contrast, the MLP performed better at the operative-level thresholds (ICDAS 3–4 vs. 0–2), where color differences are more pronounced. Although higher sensitivity values were observed for certain models within this dataset, including instances of 100% sensitivity at specific thresholds, these findings should be interpreted in the context of the study design. Given the limited sample size and the non-representative class distribution of the in vitro dataset, such estimates may not reflect performance in clinical populations. In particular, prevalence-dependent measures such as predictive values were not reported, as their inclusion could overstate clinical applicability in the absence of representative prevalence. These patterns are consistent with previous neural network studies [38–40], in which model performance tends to improve when class separation is driven by more distinct structural and optical differences. Overall, these findings suggest that different architectures may respond differently to varying degrees of color contrast within the spectrophotometric feature space [39, 40]. 

The tree-based models (RF, XGBoost, and CatBoost) showed broadly similar performance patterns, which may be related to their reliance on axis-aligned decision boundaries [41–43]. While this structure can be effective for abrupt or high-contrast features, it may be less suited to the subtle, continuous color gradients that characterize early and intermediate ICDAS stages. This tendency was reflected in both multiclass and binary classification results, where tree-based models showed lower balanced accuracy and macro-F1 scores compared with neural networks [44–48]. In binary analyses, XGBoost and CatBoost reduced assignments of sound teeth to higher ICDAS categories but were less effective in identifying early-stage lesions, while all the three tree-based models tended to prioritize specificity over sensitivity at operative threshold. These findings suggest that model structure, rather than dataset characteristics alone, may have contributed to their comparatively lower performance [41]. 

Learning-curve findings further supported this interpretation. Neural models, particularly Deep Sets, showed sustained improvement with increasing training data, whereas tree-based models exhibited early performance saturation with minimal gains beyond intermediate data fractions. **(**Table 4**)** This suggests that neural architectures may be better able to exploit additional spectrophotometric information, particularly when modelling complex, multisite relationships. In contrast, tree-based models treat each site-level measurement independently and may be less effective in capturing intra-tooth heterogeneity. Additionally, non-monotonic trends were primarily observed in tree-based models, where performance did not consistently improve with increasing data, whereas neural models showed more stable and progressive learning behaviour. These trends are likely attributable to sampling variability associated with the relatively small dataset and test set size (approximately 30 teeth), where small changes in classification outcomes can lead to noticeable fluctuations in performance metrics. This effect is likely driven by the limited number of samples within certain classes, particularly sound teeth, where a small number of misclassifications can disproportionately influence class-wise performance estimates. As a result, metrics such as sensitivity, specificity, and balanced accuracy should be interpreted with caution, particularly for underrepresented categories. Furthermore, although neural models such as Deep Sets demonstrated strong performance within this dataset, their behaviour may be influenced by the limited sample size, and performance estimates may not fully generalize to larger or more diverse datasets. In addition, class imbalance and variation in subset composition across training fractions may have contributed to this behaviour. Similar patterns have been reported in previous dental AI studies have, where three-based approaches perform will for strong predictors but are less effectively for subtle, spatially distributed features [44–46, 49, 50]. 

From a clinical perspective, the current study findings suggest that spectrophotometric CIELAB measurements may provide objective, quantitative information that complements visual assessment of occlusal caries. The models showed stronger discrimination between sound and more advanced lesions, where color differences are more pronounced, while separation between early and intermediate ICDAS categories remained more challenging. This suggests that although color-based features provide useful discriminatory information, particularly for more advanced lesions, they may not fully capture the complexity of early enamel demineralization when used in isolation. Accordingly, these approaches are best considered as adjunctive tools, with potential application as a supportive screening method for identifying more advanced lesions, while caution is required when interpreting early stage classification where color differences are more subtle.

This study has several limitations that should be considered when interpreting the findings. (1) ICDAS scoring was performed by a single calibrated examiner to ensure label consistency, which is important for supervised machine-learning, as variability in labels can adversely affect model training. While intra-examiner agreement was excellent, this approach does not account for inter-examiner variability, particularly for early ICDAS categories where visual interpretation may differ across clinicians. (2) ICDAS was used to assign labels for model training; however, as it is based on visual assessment rather than histological or micro-CT validation, some degree of uncertainty in lesion classification may be present, particularly for early lesions. Importantly, although changes in tooth color represent an early and clinically observable feature of enamel demineralization, dental caries is a multifactorial process that cannot be fully characterised by color alone. Other dental conditions, including extrinsic staining, fluorosis and developmental enamel defects, may also produce alterations in optical appearances without underlining carious pathology. In the present study, this potential confounding effect was minimised through strict inclusion criteria, excluding teeth with visible non carious defects. Accordingly, the findings should be interpreted as an evaluation of discriminatory potential of spectrophotometric color features under controlled in vitro conditions, rather than as evidence for a standalone diagnostic method.

(3) Although the test set comprised 30 teeth, this sample size reflects practical constraints associated with the use of extracted human teeth and is typical for in vitro studies; however, it may contribute to variability in performance estimates, particularly for sound teeth. Accordingly, the reported results should be interpreted with caution and require validation in larger and more diverse datasets. Additionally, the potential influence of tooth type (molars vs. premolars) on spectrophotometric measurements and model performance was not evaluated in the present study and warrants further investigation. (4) The dataset was imbalanced, with ICDAS 1–2 lesions overrepresented. This reflects the distribution of available extracted teeth and the applied inclusion criteria, rather than deliberate class selection, and may have influenced model behaviour despite the use of balanced accuracy. In addition, all measurements were obtained from under controlled in vitro conditions, which do not fully reflect the intraoral environment. Factors such as saliva, plaque, variable lighting, and probe positioning may influence color measurements and limit direct clinical translatability. The controlled design was necessary to isolate the relationship between CIELAB features and lesion severity, however, these findings require validation under clinical conditions. (5) Furthermore, prevalence-dependent measures such as positive and negative predictive values were not reported, as the dataset does not reflect the distribution of caries severity typically observed in clinical population. Given the strong dependence of these metrics on prevalence, their inclusion could overstate the clinical interpretability of the present findings. Future studies using clinically representative datasets will be required to evaluate the translational diagnostic utility of these models.

For translation into clinical practice, several steps are required. Future studies should evaluate model performance in vivo under realistic oral conditions, including variability in moisture, illumination, and operator technique. External validation on larger and more balanced datasets, inclusion of multiple examiners or consensus-based labelling, and assessment across different devices will be essential to establish robustness and generalizability. Integration with clinical workflows will also require the development of clinically interpretable decision thresholds and evaluation of real-world diagnostic impact. In addition, combining spectrophotometric color-based approaches with complementary diagnostic modalities may further enhance performance, particularly for early lesion detection. Future studies using larger datasets may also incorporate cross-validation strategies to further assess the model stability.

## Conclusion

This study suggests that spectrophotometric CIELAB measurements, when analysed using contemporary machine-learning models, may provide discriminatory information to differentiating sound, initial and moderate occlusal caries under controlled in vitro conditions. Among the evaluated algorithms, the Deep Sets model showed the most consistent multiclass ICDAS classification within this dataset, while the MLP demonstrated stronger performance for detecting moderate lesions. Tree-based models exhibited more conservative behaviour and may be less effective in capturing subtle color variations associated with early enamel changes. Overall, these findings indicate that objective color measurements may offer a quantitative, image-independent approach to caries assessment and could support future clinical and multimodal AI application.

## Supplementary Information

Below is the link to the electronic supplementary material.

Supplementary Material 1 (DOCX 997 KB) 

## Data Availability

Data supporting this study are available from the corresponding author on request.
