# Supplementary material for: Classification of occlusal caries severity using spectrophotometric CIELAB measurements and machine learning algorithms
Source: Clin Oral Investig. 2026 May 14;30(6):233. doi: 10.1007/s00784-026-06911-x (PMC13176020; doi:10.1007/s00784-026-06911-x)
Supplement: Supplementary file 1 — Supplementary Material 1 (DOCX 997 KB) [file 784_2026_6911_MOESM1_ESM.docx]

**Supplementary File 1**

**SP Figure 1:** Visual representation of this experimental setup for ICDAS labelling and spectrophotometric data collection

**
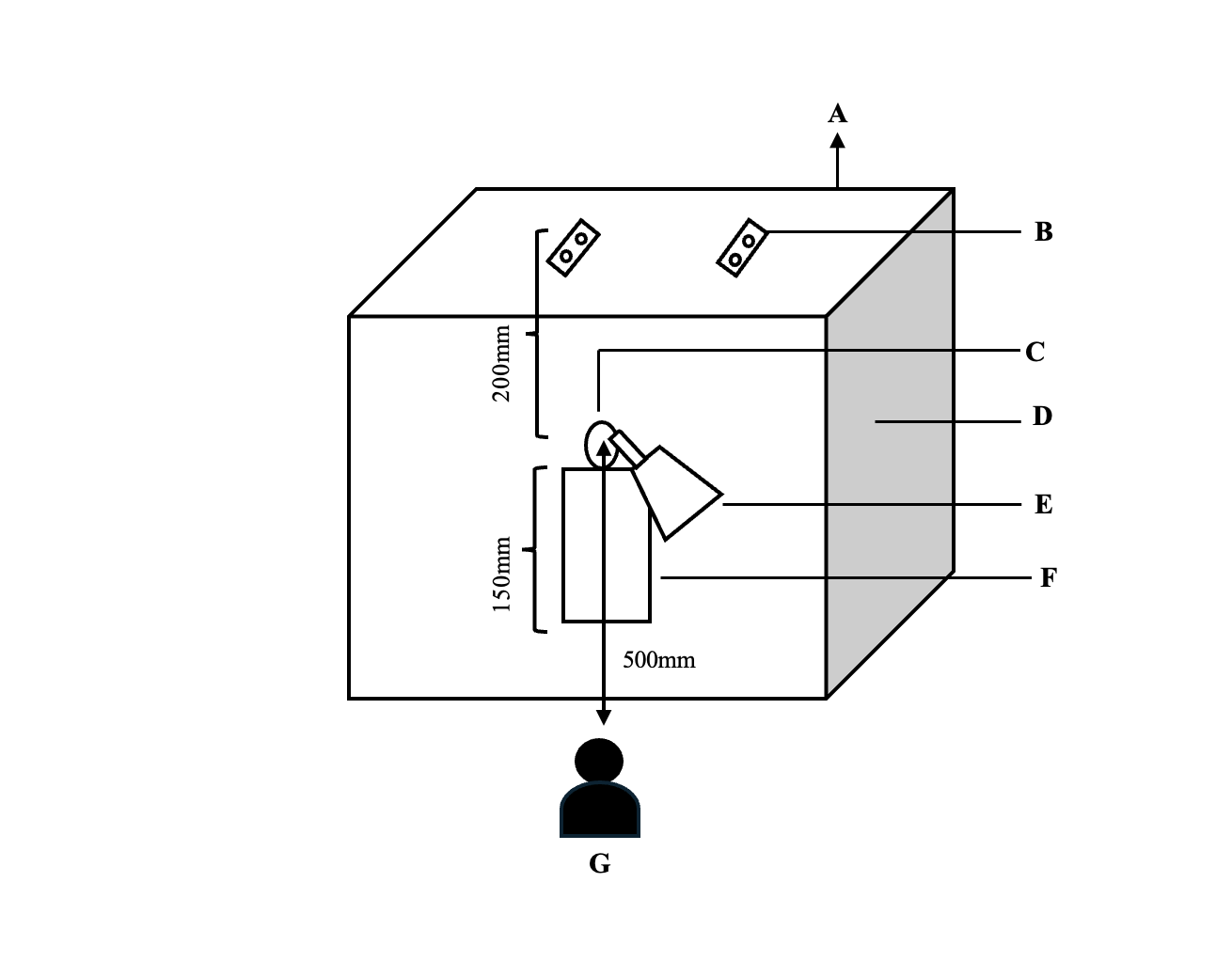
**

A)The PhotoBox; B) In-built LED lights; C) occlusal surface of the tooth facing the examiner; D) Gray background; E) Spectrophotometer prob placed on the occlusal surface of the teeth; F) Platform; G) Examiner

**SP Figure 2:** Representative images of ICDAS categories (Sound, initial lesion and moderate lesion


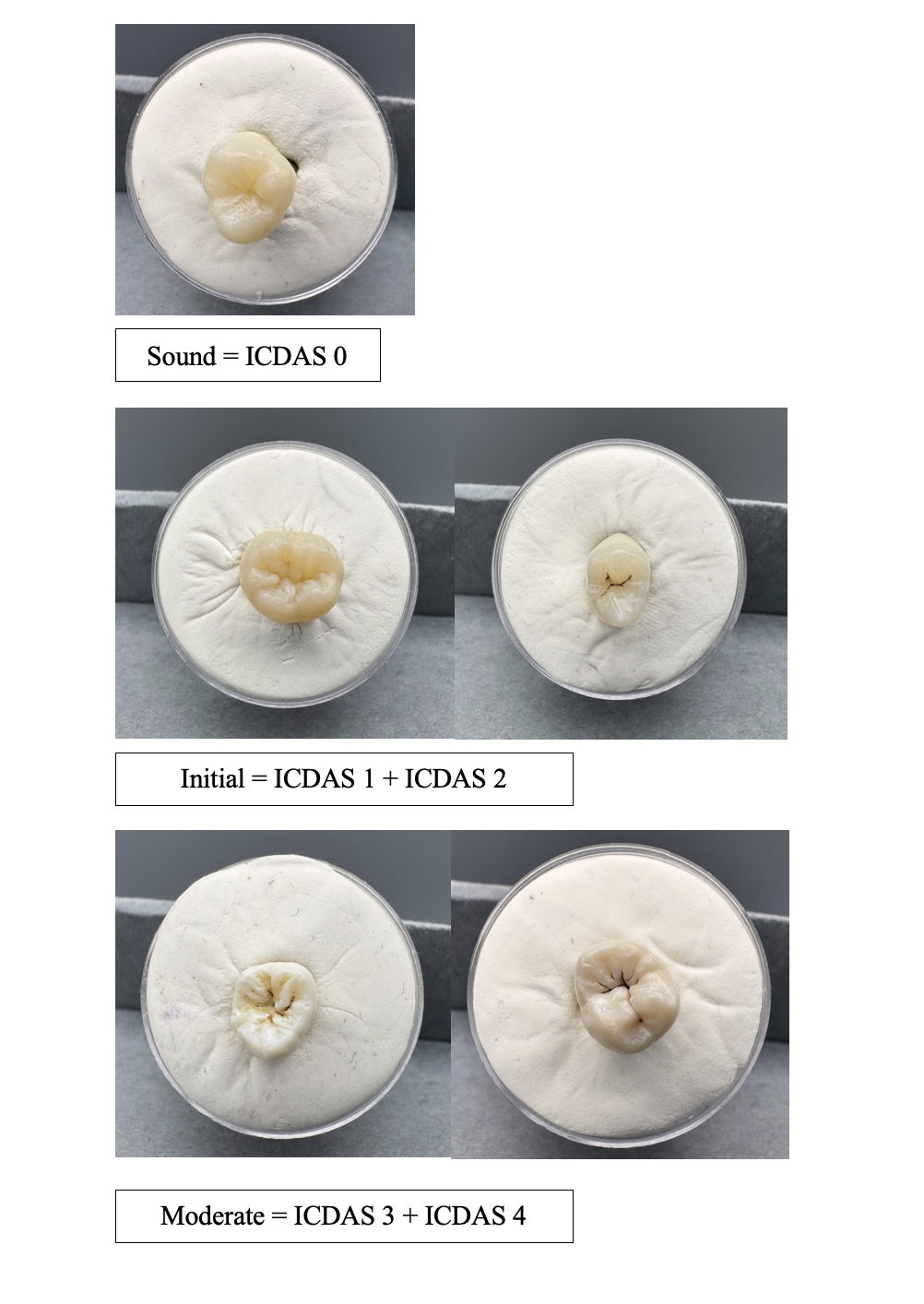


**SP Table 1:** Specifications, training procedures, and hyperparameter settings of the machine learning models
**Tree-based models**

- **Random Forest (RF):** 100 trees with bootstrap aggregation, random feature selection (max_features = “sqrt”) and class-balanced sampling. Tree depth was left unconstrained to allow full hierarchical partitioning of the engineered color features.
- **XGBoost:** shallow trees (max_depth = 5), learning rate = 0.03, L2 regularization (reg_lambda = 5.0), and subsampling = 0.8, using a multiclass soft probability objective with early stopping.
- **CatBoost:** 200 boosting iterations, tree = 6, learning rate = 0.05, and L2 leaf regularization, with balanced class weights and a multiclass loss function.

**Multiplayer perceptron (MLP)**

The network consisted of two 256 units ReLU layers followed by a 128 unit layer. Gaussian noise (σ = 0.05) and dropout (0.4) were used to reduce overfitting. A masked mean pooling layer aggregated the five point-level representations into a single tooth level embedding, followed by a Softmax output for the three ICDAS categories.

**Deep Sets model with attention pooling**
Each measurement point was processed through two 128 unit ReLU layers with dropout (0.3) to form a point level embedding. These were aggregated using an attention-based pooling mechanism to generate a permutation-invariant tooth-level representation. The classification head comprised a 128 unit dense layer with dropout (0.3) and a Softmax output for the three ICDAS categories.

Hyperparameter tuning was conducted using a two-stage process. Initial core settings were identified through exploratory tuning, after which parameters with the greatest influence on model behaviour, such as the number of trees/iterations, aggregation settings, decision thresholds, and calibration exponents, were optimized using a structured grid search on the internal validation set. All tuning was confined strictly to the training partition to prevent information leakage.

**SP Table 2: Derivation of color features and data preprocessing**
Each tooth was treated as a single analytical unit comprising five spectrophotometric measurement sites. Baseline color was defined as mean L*, a*, b* values across the five sites. Site specific deviations were derived for each site, including chroma (C = √(a² + b²)) and hue angle (h = tan⁻¹(b/a)), along with their intra-tooth deviations (ΔC, Δh). Overall color differences (ΔE) were obtained using CIE76 formula [ΔE = √((ΔL)² + (Δa)² + (Δb)²)], calculated relative to (i) mean color of each tooth and (ii) a reference ‘healthy color centroid’, defined as the mean L*, a* and b* values of ICDAS 0 teeth within the training set. Site-specific differences from the overall tooth color were quantified using ΔL, Δa, and Δb, calculated as residual differences from the mean tooth color.

Categorical variables, tooth type and measurement site index, were incorporated to account for anatomical and spatial variation. For tree-based models (RF, XGBoost and CatBoost), these variables were one hot encoded. In the Deep Sets model, they were represented as learnable embeddings to capture relationships across the measurement sites. The MLP model processed site-level numerical features and incorporated them using a masked mean pooling mechanism to generate a single tooth level representation. trained using only standardized numerical features without categorical inputs. These preprocessing strategies ensured that all models received appropriately structured inputs while preserving site-level information, enabling the extraction of clinically relevant color patterns associated with caries severity.
